# Tetrahedral framework nucleic acids‐based delivery promotes intracellular transfer of healing peptides and accelerates diabetic would healing

**DOI:** 10.1111/cpr.13279

**Published:** 2022-07-09

**Authors:** Shiyu Lin, Qi Zhang, Songhang Li, Xin Qin, Xiaoxiao Cai, Huiming Wang

**Affiliations:** ^1^ Stomatology Hospital, School of Stomatology, Zhejiang University School of Medicine, Zhejiang Provincial Clinical Research Center for Oral Diseases, Key Laboratory of Oral Biomedical Research of Zhejiang Province Cancer Center of Zhejiang University Hangzhou China; ^2^ Department of Implant Dentistry, Shanghai Ninth People's Hospital Shanghai Jiao Tong University School of Medicine Shanghai China; ^3^ College of Stomatology Shanghai Jiao Tong University Shanghai China; ^4^ National Center for Stomatology, National Clinical Research Center for Oral Diseases Shanghai China; ^5^ Shanghai Key Laboratory of Stomatology Shanghai China; ^6^ State Key Laboratory of Oral Diseases, National Clinical Research Center for Oral Diseases, West China Hospital of Stomatology Sichuan University Chengdu China

## Abstract

**Objectives:**

Peptide‐based therapeutics are natural candidates to desirable wound healing. However, enzymatic surroundings largely limit its stability and bioavailability. Here, we developed a tetrahedral framework nucleic acids(tFNA)‐based peptide delivery system, that is, p@tFNAs, to address deficiencies of healing peptide stability and intracellular delivery in diabetic wound healing.

**Materials and Methods:**

AGEs (advanced glycation end products) were used to treat endothelial cell to simulate cell injury in diabetic microenvironment. The effects and related mechanisms of p@tFNAs on endothelial cell proliferation, migration, angiogenesis and ROS (reactive oxygen species) production have been comprehensively studied. The wound healing model in diabetic mice was photographically and histologically investigated in vivo.

**Results:**

Efficient delivery of healing peptide by the framework(tFNA) was verified. p@tFNAs helped overcome the angiogenic obstacles induced by AGEs via ERK1/2 phosphorylation. In the meantime, p@tFNA exhibited its antioxidative property to achieve ROS balance. As a result, p@tFNA improved angiogenesis and diabetic wound healing in vitro and in vivo.

**Conclusions:**

Our findings demonstrate that p@tFNA could be a novel therapeutic strategy for diabetic wound healing. Moreover, a new method for intracellular delivery of peptides was also constructed.

## Graphical abstract

Tetrahedral framework nucleic acids(tFNA)‐based peptide delivery system enhances the stability and intracellular transport efficiency of healing peptides. Combined with the antioxidant property of tFNA, the delivery system improves angiogenesis and diabetic wound healing via promoting the angiogenic behaviors via ERK1/2 phosphorylation and achieving ROS balance in diabetic micro‐environment.

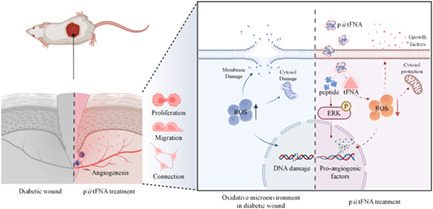

## 1 INTRODUCTION

Unhealed wound ulcers induced by poorly controlled diabetes put patients at risk of infection and amputation. With deficient angiogenesis in the pathological characteristics, few newly formed capillaries are observed in diabetic ulcers.^1^, ^2^ Attenuation of angiogenesis directly leads to reduced delivery of oxygen and nutrients, which play a vital role in the progression of the proliferative phase.^2^, ^3^ In addition to the defective granulation tissue, the communications of inflammatory components and growth factors also decrease.

Deficient angiogenesis is closely related with oxidative microenvironments formation induced by extracellular and intracellular accumulation of advanced glycation end products (AGEs).^4^, ^5^ On one hand, AGEs bind to receptors on the endothelial cell surface and subsequently induce intracellular overproduction of reactive oxygen species (ROS). One the other hand, AGEs deleteriously damage the crosslink of extracellular matrix. The injured crosslinking of extracellular matrix proteins leads to abnormal binding with proteoglycans and collagenase, which are responsible for the imbalance of reactive oxygen species generation.^4^, ^6^ In turn, the elevation of oxidative stress expedites AGEs deposition and form a vicious cycle.^7^, ^8^ In the deteriorating microenvironment, oxidative stress leads to membrane phospholipid peroxidation, proteins oxidation, and DNA damage. In addition, the abnormal elevation in proteolytic activity also leads to growth factor degradation and subsequently attenuates angiogenesis in wound healing.^4^, ^7^, ^8^ Based on the complex and variable physiological environment in vivo, it is still a challenge to design a delivery system for the changes of metabolic state. Various studies focused on solving the diabetic micro‐environment.^9^, ^10^

Current treatment of diabetic wound healing includes blood glucose control, surgical debridement, off‐loading, attention to oxidation, and vascular reconstruction. Peptide‐based therapeutics are natural candidates to target microbial infections, diabetes and vascular diseases due to their high bioactivity and selectivity.^11^, ^12^, ^13^ Short peptides take part in the transmission of biological information and subsequently maintain cellular home‐stasis. Various studies have explored the role of peptides as biological regulators, which maintain major physiological functions and play an important role in the complex steady‐state process chain leading to cell, tissue and organ aging.^14^, ^15^ Studies have shown that REGRT healing peptide could enhance cell migratory ability through promotion of matrix metalloproteinases via ERK signaling pathway.^16^ Nevertheless, current peptide drugs are developed toward extracellular targets such as cell membrane receptors and secreted proteins, with only a small percentage of intracellular usage. Intracellular delivery of peptides brings hope to target former undruggable sites and will expand the category of peptide‐based therapeutics.^17^, ^18^ Additionally, enzymatic surroundings largely limit the stability, affect the bioavailability and shorten the half‐life of peptides.^17^, ^19^ It is necessary to explore a facile and robust solution to penetrate physiological barriers and protect cytosolic peptides from extracellular and intracellular interference.

Nanoparticles acts as a multifunctional drug delivery platform to protect against enzyme degradation, release drug accurately, penetrate physiological membranes, and deliver the drug to specific sites.^20^, ^21^ However, improper immune and inflammatory responses might occur in nanoparticles with poor biocompatibility. FNA, with advantages in aspects of drug loading, multi‐site modification, and low‐immunogenicity, has emerged as a promising candidate to deliver various cargos, such as nucleic acids and proteins.^22^, ^23^, ^24^, ^25^

The rapid development of nucleic acid research breeds a diversity of FNAs applied in drug delivery, medical engineering, and disease diagnosis.^22^, ^23^, ^26^ FNA structures are characterized by flexible joints, abundant incubation sites and less spatial confinement to the cargos. The dimensional framework structure provides internal hollow space and exterior adsorption sites for the attachment of drug molecules and compounds. In the meanwhile, the inherent properties of better cellular uptake and framework protection facilitate delivery with enhanced resistance to surrounding enzymes.^27^, ^28^ As a result, the conjunctions of FNA and compounds (such as lipids, peptides, and proteins) are designed as delivery systems.^22^, ^23^, ^26^ The adhesion of lipids, peptides and proteins onto FNA breeds the prevention of FNA from complex surroundings and protects the antioxidant ability of tFNA.

Based on the above, we fabricate a tFNA‐based REGRT healing peptide delivery system to enhance transportation with less loss into cells, which may overcome the instability and uptake limitations of peptides. tFNA provides a frame foundation and hollow internal space for electrostatic adsorption of healing peptides, while the attachment of peptide covers outside to wrap around tFNA. The tFNA frame facilitates the uptake and stability of peptides, while the peptide protects tFNA from DNase. The interaction addresses the other's deficiency while giving full play to its inherent property.

## 2 MATERIALS AND METHODS

### 2.1 Synthesis of p@tFNA

The synthesis steps of p@FNA are as follows. First, four types of single‐stranded DNA were synthesized according to the pre‐designed base sequences (Table 1). Subsequently, equimolar ratios of the four types of single‐stranded DNA were added to TM buffer and denatured in a thermal cycler at 95°C for 10 min, followed by complementary base pairing at 4°C for at least 30 min. Healing peptide (REGRT) solutions of different concentrations were added at 1:1 (volume ratio) and mixed well. After shaking for 30 s and quiescence for 30 min, solutions were collected, sterilized by filtration through a 0.22 μm filter.

**TABLE 1:** Sequences for tFNA fabrication

	5′–3′ base sequence
Strand1	ATTTATCACCCGCCATAGTAGACGTATCACCA
	GGCAGTTGAGACGAACATTCCTAAGTCTGAA
Strand2	ACATGCGAGGGTCCAATACCGACGATTACAG
	CTTGCTACACGATTCAGACTTAGGAATGTTCG
Strand3	ACTACTATGGCGGGTGATAAAACGTGTAGCAA
	GCTGTAATCGACGGGAAGAGCATGCCCATCC
Strand4	ACGGTATTGGACCCTCGCATGACTCAACTGCC
	TGGTGATACGAGGATGGGCATGCTCTTCCCG

### 2.2 Characterization of p@tFNA

The successful preparation of p@tFNA was verified by polyacrylamide gel electrophoresis (PAGE) at 60 V for 80 min. The morphology of p@tFNA was characterized by transmission electron microscopy (TEM). Hydrated particle size and zeta potential of p@tFNA were examined using Zeta sizer Nano ZS90 dynamic light scattering (DLS).

### 2.3 Encapsulation efficiency and loading capacity

p@tFNA was added to ultrafiltration centrifuge tubes and centrifuged at 6000 rpm for 5 min. Peptides not bound to FNA were filtered out. The absorbance of the peptide solution was measured by UV spectrophotometer and a standard curve (205 nm) was established. Peptide encapsulation efficiency and loading content for this method were calculated according to the formula shown below:

(1) $$\text{Encapsulation}\;\text{efficiency}=\frac{\text{Total}\;\text{peptide}-\text{Uncombined}\;\text{peptide}}{\text{Total}\;\text{peptide}}$$

(2) $$\text{Loading}\;\text{content}=\frac{\text{Total}\;\text{peptide}-\text{Uncombined}\;\text{peptide}}{\text{tFNA}}\times 100\%$$

#### 2.3.1 Enzyme stability of p@tFNA (trypsin and DNase)

As to the enzyme stability of peptide, equal amount of peptide and p@FNA were treated with trypsin and incubated in 37 °C for 1.5, 3, and 6 h. The remaining amount of peptide was measured by UV spectrophotometer (205 nm). As to enzyme stability of tFNA, equal amount of tFNA and p@FNA were treated with DNase I for 1 min. After neutralization, samples were examined by 6%PAGE at 60 V for 80 min.

#### 2.3.2 Simulated diabetic environment

HUVECs under diabetic microenvironment were induced by incubation with 200 μg/ml AGEs (Bioss, Beijing, China) for 24 h. Cells were then treated with peptides or p@FNA (125 nM) for subsequent experiments.

#### 2.3.3 Cell uptake of p@tFNA

5‐FAM labeled peptide was used for observation. Endothelial cells were starved for 12 h and treated with healing peptide and p@FNA for 6 h. After fix with paraformaldehyde, cells were treated with phalloidin and DAPI to stain cytoskeleton and nuclei, respectively. Finally, images were collected by confocal microscopy.

#### 2.3.4 Cell migration

After 24 h pre‐incubation with AGEs, cells were scratched with a 20 μl pipette tip and subsequently treated with peptide or p@FNA. Images were taken after 24 and 36 h. Wound healing analysis was performed with Image J software.

#### 2.3.5 Cell viability

The viability of endothelial cells under different treatments was tested by CCK‐8 assay. Briefly, 10 μl of CCK‐8 solution and 100 μl of fresh medium were added to each well of a 96‐well plate and incubated for 1 h. Absorbance at 450 nm were collected with a microplate reader.

#### 2.3.6 Tube formation assay

The formation of blood vessel‐like structures in vitro was observed by tube formation assay. Cells were pre‐incubated with AGEs for 24 h and subsequently treated with peptide or p@FNA for another 24 h. Subsequently, 100 μl of HUVEC (1–1.5 × 10^5^ cells/ml) were seeded on Matrigel (Corning, NY) in 96‐well plates. Images were taken after 6 h. Statistical analysis of tube formation was performed by Image J.

#### 2.3.7 Real‐time polymerase chain reaction (PCR)

The gene expression levels of VEGFA, TGF‐β, FGF, PDGF, MMP‐2, and MMP‐9 were detected by real‐time quantitative PCR (q‐PCR). The sequences of primers are shown in Table 2. Briefly, total mRNA in endothelial cells was extracted by TRIzol (Thermo Fisher, MA). The cDNA of interest was amplified as follows: 95°C for 30 s, 40 cycles (95°C for 5 s, 60°C for 34 s).

**TABLE 2:** Sequences of primers for q‐PCR

Gene		5′–3′ base sequence
*VEGFA*	F	ATCGAGTACATCTTCAAGCCAT
	R	GTGAGGTTTGATCCGCATAATC
*TGF‐β*	F	GCGAAGCTGACCTGGAAGAG
	R	TGCTGAGGTATCGCCAGGAA
*FGF2*	F	CATCAAGCTACAACTTCAAGCA
	R	CCGTAACACATTTAGAAGCCAG
*PDGFB*	F	GATCCGCTCCTTTGATGATCTC
	R	GGTCATGTTCAGGTCCAACTC
*MMP2*	F	ATTGTATTTGATGGCATCGCTC
	R	ATTCATTCCCTGCAAAGAACAC
*MMP9*	F	CAGTACCGAGAGAAAGCCTATT
	R	CAGGATGTCATAGGTCACGTAG

#### 2.3.8 Western Blotting

The expression of β‐actin, VEGFA, MMP‐9, ERK1/2 and p‐ERK1/2 was detected by western blotting. The total protein of the sample was extracted by whole cell lysis method. The target protein was separated by 10% SDS‐PAGE and transferred to PVDF membrane. The bands were blocked in 5% BSA for 1 h and then incubated with primary antibodies against β‐actin (1:5000, PMK058S, BioPM, Wuhan, China), VEGFA (ab214424, Abcam, Cambridge, UK), MMP‐9 (ab76003, Abcam, Cambridge, UK), ERK1/2 (1:1000, CST, #4370, MA) and p‐ERK1/2 (1:2000, CST, #4370, MA) overnight at 4°C. The next day, PVDF membranes were rewarmed and incubated with secondary antibodies for 1 h. Bands were washed three times with TBST. The final exposure was performed in a Bio‐Rad chemiluminometry.

#### 2.3.9 Total antioxidant capacity test

The antioxidant capacity of tFNA, peptide and p@tFNA were detected by the CheKine™ Total Antioxidant Capacity (TAC) Assay Kit (KTB1500, Abbkine, CA, USA). Briefly, 150 μl of substrate diluent and 15 μl of substrate were premixed, then 15 μl of reaction buffer and 10 μl of samples were added. Incubate for 5 min at room temperature. Measure the absorbance at 593 nm with the microplate reader.

#### 2.3.10 Hydroxyl free radical scavenging capacity test

The hydroxyl radical scavenging capacity of tFNA, peptide and p@tFNA was tested by CheKine™ Hydroxyl Free Radical Scavenging Capacity Assay Kit (KTB1091, Abbkine, CA, USA). Briefly, we mixed 20 μl chromogen A, 40 μl assay buffer and 20 μl chromogen B. Then, we added 20 μl of sample, 20 μl H_2_O_2_, and incubated at 37°C for 60 min after mixing. Absorbance was measured at 536 nm.

#### 2.3.11 ROS level detection

DCFH‐DA probe was used to detect the intracellular reactive oxygen species level. After pre‐incubation with peptide or p@tFNA for 12 h, cells were treated with H_2_O_2_ for another 24 h. Then DCFH‐DA probe was added and incubated for 25 min. Subsequently, the DCFH‐DA solution was discarded and cells were rinsed with PBS. After digestion with trypsin, the cell suspension fluorescence was detected at 488 nm by a microplate reader.

#### 2.3.12 Immunofluorescence staining

After adding AGEs for 24 h, the peptides and p@tFNA were added and incubated for another 24 h. Subsequently, cells were fixed with paraformaldehyde (4% [w/v]) and treated with Triton X‐100 (0.5%). After block and incubation with HO‐1 primary antibody (ab68477, Abcam, Cambridge, UK), the samples were incubated with fluorescent secondary antibodies. Cytoskeleton and nuclei were subsequently stained with phalloidin and DAPI, respectively. Finally, the images were collected and observed by a confocal microscope (Nikon, Tokyo, Japan).

#### 2.3.13 Diabetic type II mouse skin defect model

Diabetic type II full‐thickness wounds model was established in db/db mice (female, 8w). The animal experiment was approved by the ethics committee of Zhejiang University. Mice were randomly divided into 3 groups: control group (physiological saline), peptide group and p@tFNA group. 8 mm full‐thickness skin defects were prepared in the dorsal skin of mice using a skin biopsy. Drug injections were performed at four sites on the edge of the defect every 3 days after defect preparation. Defect areas were recorded and measured at 1st, 5th, 10th, and 15th day from the first processing. Mice were euthanized on day 15 and dorsal skin was collected for histological staining.

#### 2.3.14 Statistics

Statistical analyses were performed with two‐tailed t‐tests or one‐way analysis of variance (GraphPad Prism 7, San Diego). Data are presented as the means ± SD. *p* < 0.05 was considered as statistically significant.

## 3 RESULTS

### 3.1 Synthesis of p@tFNA and loading of peptides

**FIGURE 1:**
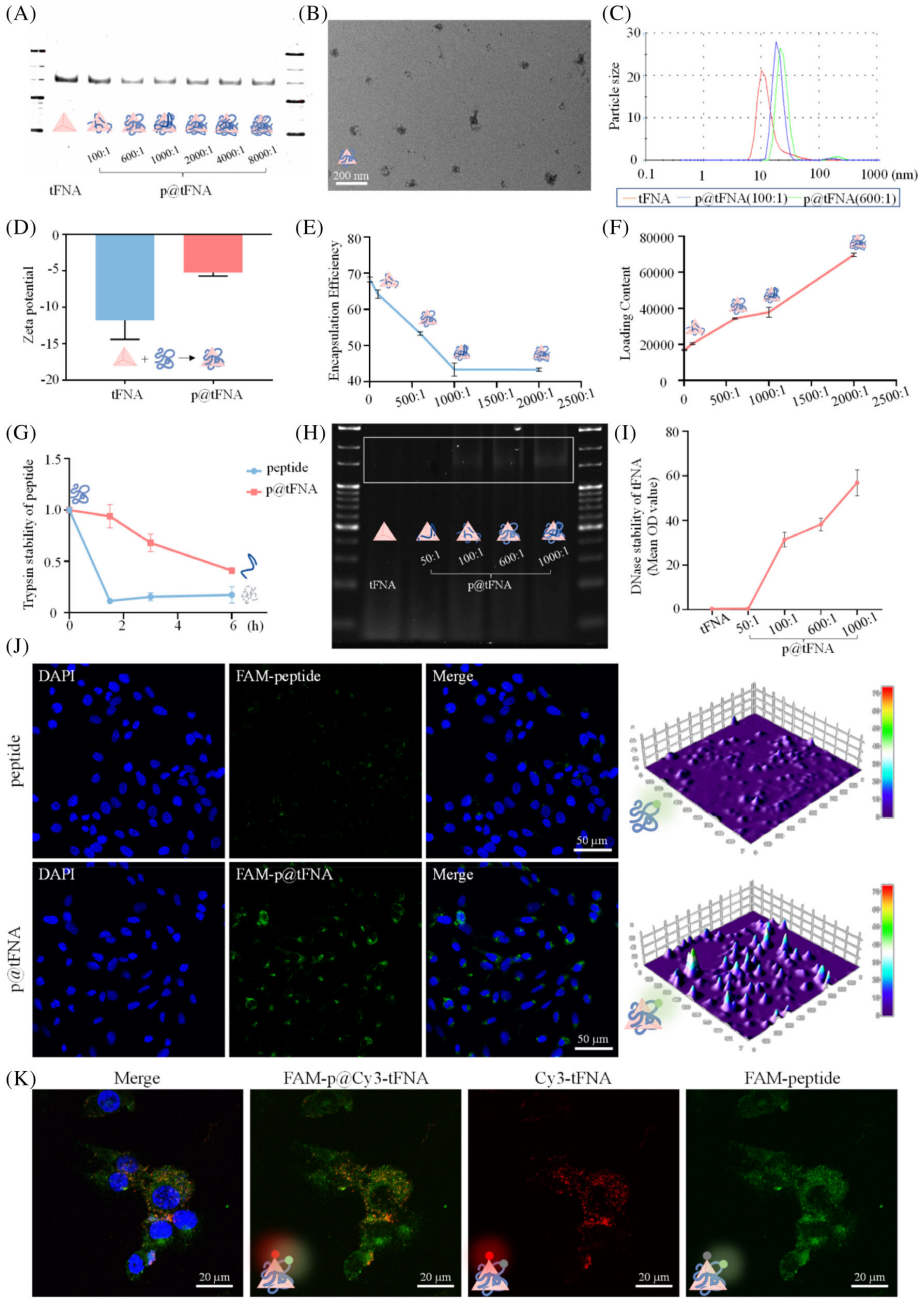
Fabrication and characterization of tFNA and p@tFNA. (A) PAGE detection and schematic diagram showing the synthesis and composition of tFNA and p@tFNA. (B) TEM image showing the morphology of p@tFNA. (C,D) Size and zeta potential of tFNA and p@tFNA. (E) Ultraviolet–Visible absorbance for encapsulation efficiency curve of healing peptides loaded on tFNA. (F) Loading content of healing peptides loaded on tFNA. (G) Stability detection of peptides with treatment of trypsin for 1.5, 3, and 6 h. (H,I) PAGE detection and semi‐quantification for stability of tFNA and p@tFNA in DNase I environment. (J) Cellular uptake of FAM‐labeled peptides and FAM‐labeled p@tFNA. (K) Cellular uptake of p@tFNA composed of FAM‐labeled peptide and Cy3‐labelled tFNA. Data are presented as mean ± SD (*n* = 4). Statistical analysis: *p < 0.1; **p < 0.01.

We employed PAGE and TEM to intuitively reflected the molecular weight and morphology of p@tFNA in Figure 1A,B. Regarding other characteristics such as size and zeta potential, the sizes of the complexes increased with increasing peptide concentration (Figure 1C). Sizes of the p@tFNA(100:1) and p@tFNA(600:1) proportions attached to approximately 18.06 ± 1.19 and 21.31 ± 1.36 nm, respectively, compared with the 11.19 ± 1.86 nm of simple tFNA. In addition to magnifying the size, the absorption of healing peptides also attenuated the negative charge of tFNA from −11.78 ± 2.15 mV to −5.24 ± 0.40 mV (Figure 1D).

UV spectrum scanning was taken to further assess the encapsulation efficiency and loading content between peptide and tFNA. As shown in Figure 1E,F, corresponding to the rising peptide proportion, the encapsulation efficiency decreased while the loading content increased. By taking these two factors into consideration, the combination of peptide and tFNA achieved best effect at a proportion of 600:1, with encapsulation efficiency percentage and loading content OD value, respectively, reaching 53.32 ± 0.39% and 34352.75 ± 261.45). Considering the satisfactory construction of p@tFNA with suitable encapsulation and less interfere with the intrinsic property, we chose p@tFNA (600:1) as an appropriate ratio for further experiments.

### 3.2 Developed enzyme stability and cellular membrane crossing of peptides

From another point of view, tFNA acted as an inner “framework” for effectively and completely delivering peptides for cytosol application. Peptides (positive charge) attached to the framework (negative charge) due to electrostatic adhesion. To explore whether tFNA could act as a “framework” to absorb, support and protected encapsulated peptides from enzyme environment, we adopted trypsin and UV spectrum scanning to detect the residual peptides in simple peptide group and p@tFNA group (peptides absorbed onto the framework DNA). Curves in Figure 1G proved the support and protection role of tFNA in peptide stability. Compared with the rapid decomposition of simple peptides during 1.5 h, peptides attaching on the framework DNA exhibited better resistance to trypsin. The residual peptides percentages remained 93.92 ± 9.28% at 1.5 h, 68.09 ± 6.99% at 3 h and 40.89 ± 1.72% at 6 h, respectively.

Similarly, DNase I and PAGE were taken to detect whether the adsorption of peptides could protect tFNA from the surrounding environment. Bands in Figure 1H revealed that the combination with peptides helped prevent tFNA from DNase I degrading. Higher OD values of DNA samples analyzed in Figure 1I could be detected with increasing wrap of peptides. The protective effect was more obvious at the 600:1 and 1000:1 proportion than that at 50:1.

In addition, the cellular uptake of peptides with the help of tFNA as a drug carrier was also investigated by 5‐FAM‐labeled peptides and laser confocal microscopy. As seen in the Figure 1J, attached to tFNA, more 5‐FAM‐labeled peptides could be absorbed and observed in the cytoplasm after incubation for 6 h. Further combination of FAM‐labeled peptides and Cy3‐labeled tFNA in Figure 1K indicated the intracellular delivery role of tFNA.

### 3.3 Promotion of migration, proliferation, and tube formation

**FIGURE 2:**
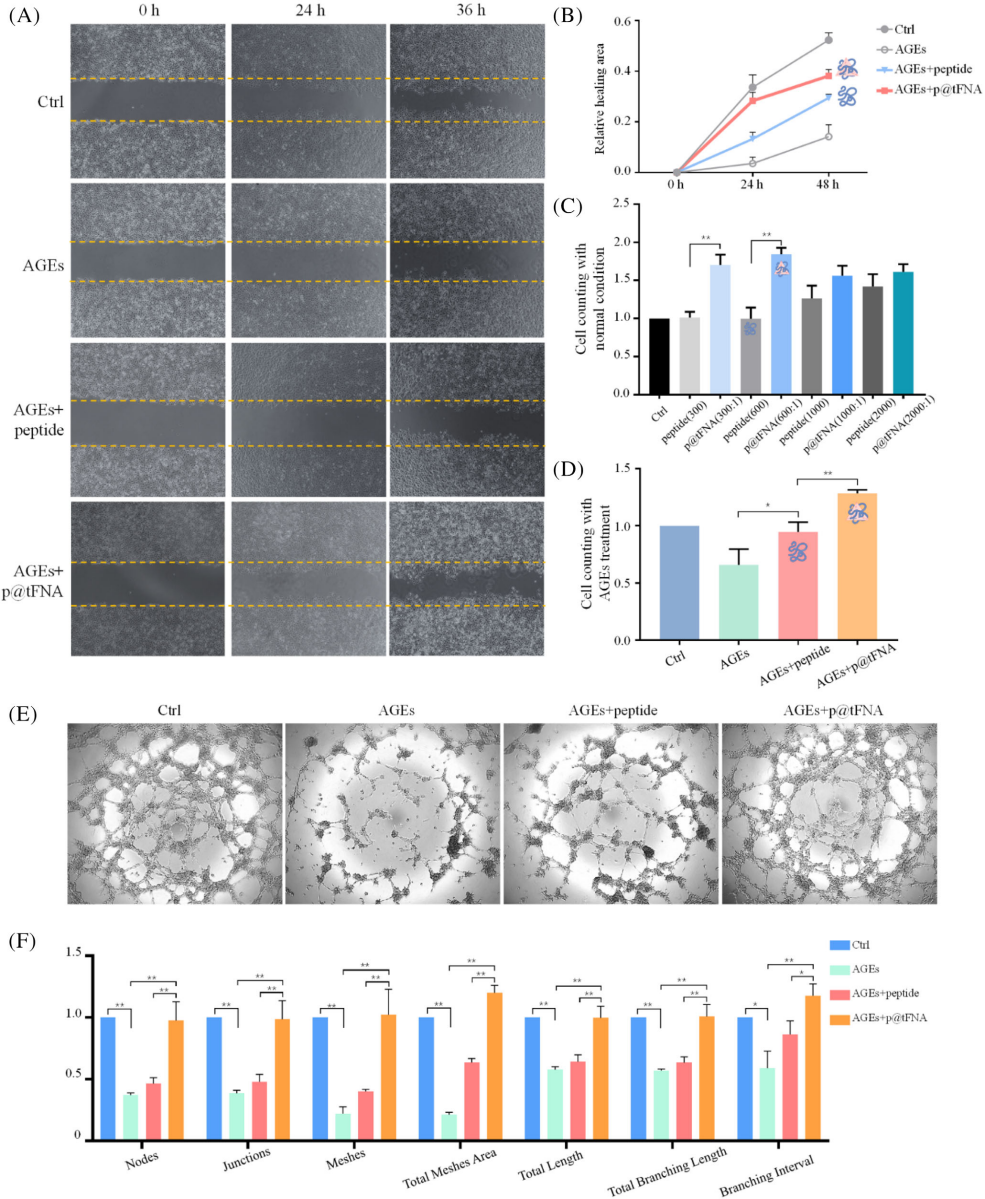
p@tFNA enhanced migration, proliferation, and vascularization ability of endothelial cells via ERK1/2 signal. (A,B) Wound healing closure assay and semi‐quantification analysis of AGEs‐treated HUVECs with peptide and p@tFNA treatment. (C) Cell counting assay examining proliferation of endothelial cells in control group, peptide group and p@tFNA (300:1, 600:1, 1000:1, and 2000:1) groups. (D) CCK8 detection of cell proliferation in control group, AGEs group, AGEs+peptide group and AGEs+p@tFNA (600:1) group. (E) Tube formation assay for evaluating vascularization after incubation of AGEs and further treatment of peptide or p@tFNA. (F) Analysis of tube nodes, junctions, meshes, total meshes area, branching lengths and branching intervals of formed vessels. Data are presented as mean ± SD (*n* = 3). Statistical analysis: *p < 0.1; **p < 0.01.

The migration speed of ECs (Figure 2A,B) in AGEs inducing diabetic environment were found slower than those in the control group. The average healing areas were only 3.52% at 24 h and 14.11% at 36 h. As to the wound in the peptide group, the average healing areas were up to 13.21% at 24 h and 29.42% at 36 h. Compared with simple peptide, peptides delivered by tFNA achieved better healing effects up to 28.28% at 24 h and 38.14% at 36 h.

We also adopted cell counting kit (CCK‐8) to assess the biological effect of different peptide‐tFNA proportions. Results in Figure 2C showed that the HUVECs in p@tFNA (300:1) and p@tFNA (600:1) groups exhibited better cell viability than those treated with simple peptides. However, only slight changes could be observed in proportions of 1000:1 and 2000:1. Combined with results of encapsulation efficiency and enzyme stability, we chose 600: 1 (peptide:tFNA) during subsequent works. The histogram in Figure 2D indicated the positive role of p@tFNA in a diabetic microenvironment induced by AGEs. Compared with control group, cell viability reduced to 0.66‐fold in the AGEs environment and increased to 0.94‐fold in peptide group and 1.28‐fold p@tFNA group.

Apart from cell proliferation, migration of endothelial cells, subsequent organization of tubular structures is considered the direct representation and crucial process during angiogenesis.^29^ To intuitively observe the effect of p@tFNA on the angiogenesis of wound healing, a cell tube formation assay was performed under AGEs‐induced conditions (Figure 2E). HUVECs treated with p@tFNA exhibited better tube formation in the Matrigel than those treated with simple peptides. A higher quantity and density of capillary‐like networks could be observed. Nodes, junctions and meshes were identified with total mesh area, total length, branching length and branching intervals calculated in Figure 2F. These angiogenesis‐associated elements were attenuated by AGEs and up‐regulated after delivery of healing peptide. The nodes, junctions and meshes of the newly formed vessel‐like structures in the p@tFNA group was 2.10‐fold, 2.05‐fold, and 2.55‐fold that of the structures stimulated by peptides. Similar trends could be found in the total mesh area, total length, branching length and branching intervals. These indicators facilitated by p@tFNA were 1.89‐fold, 1.55‐fold, 1.59‐fold, and 1.36‐fold of the simple peptide group, respectively.

### 3.4 Proangiogenic genes, proteins, and ERK1/2 signaling involved in p@tFNA Induction

**FIGURE 3:**
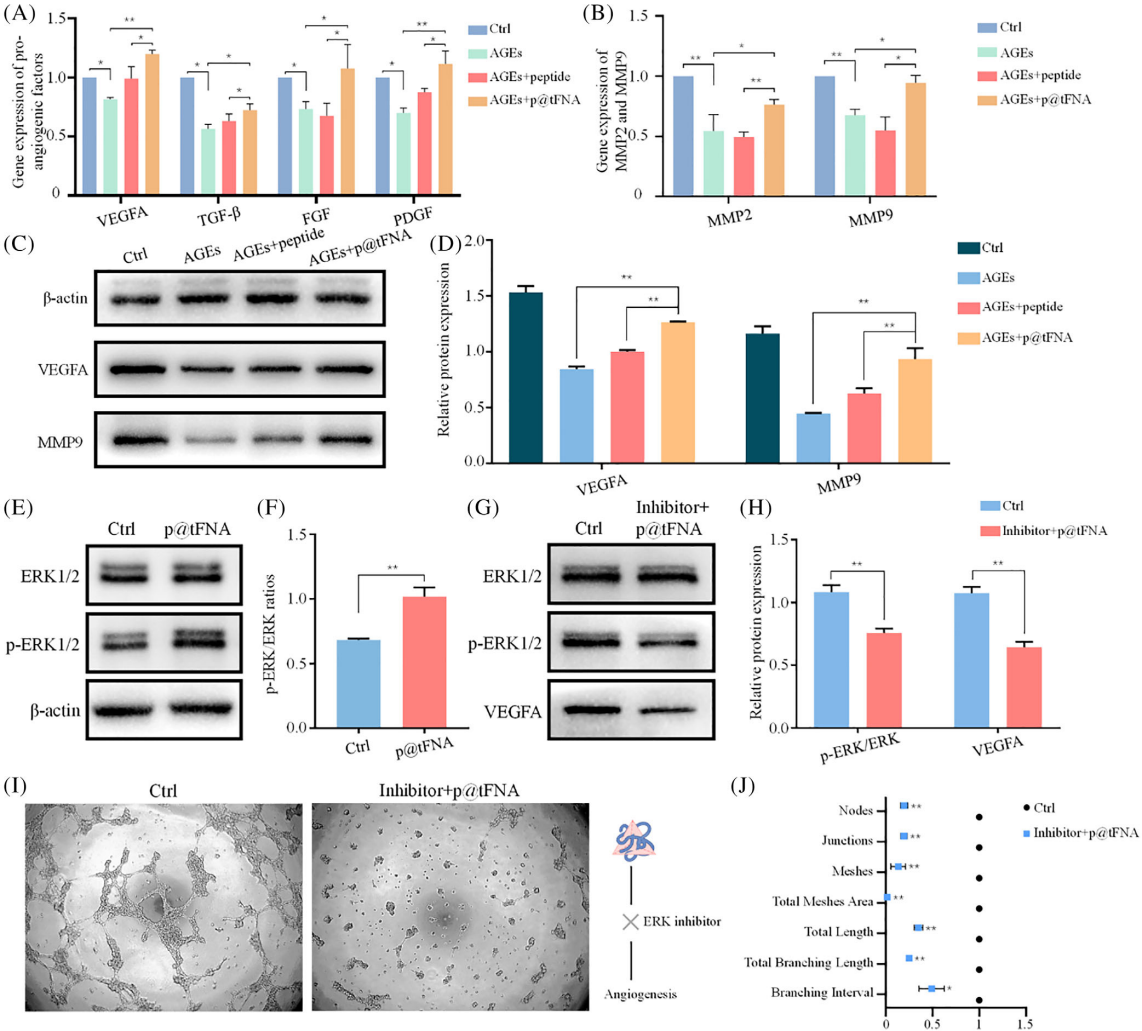
Expressions of pro‐angiogenic factors were up‐regulated by p@tFNA through ERK1/2 phosphorylation. (A,B) Gene expression of VEGFA, TGF‐β, FGF and PLGF, MMP2 and MMP9 in control group, AGEs group, AGEs+peptide group and AGEs+p@tFNA (600:1) group. (C,D) Western blotting result of the VEGFA and MMP9 expression and corresponding statistical analysis. (E,F) Western blotting result of ERK1/2 phosphorylation after p@tFNA treatment. (G,H) Western blotting result of ERK1/2 phosphorylation and VEGF expression after signal pathway inhibition and corresponding analysis. (I) Tube formation assay after ERK1/2 inhibition. (J) Analysis of tube nodes, junctions, meshes, total meshes area, branching lengths and branching intervals of formed vessels after ERK1/2 inhibition. Data are presented as mean ± SD (*n* = 3). Statistical analysis: *p < 0.1; **p < 0.01.

Multiple mechanisms involve in the reduced vessel growth. Molecular level variations including proangiogenic genes, proteins and potential signal pathway were further detected to explain the above‐mentioned angiogenic cellular behaviors. As indicated in Figure 3A,B, gene expression of VEGFA, TGF‐β, FGF2, PDGFB, MMP2, and MMP9 was elevated to varying degrees in the simple peptide group. In the p@tFNA group, the expression of VEGFA, TGF‐β, FGF2, PDGFB, MMP2, and MMP9 was further facilitated up to 1.21‐, 1.14‐, 1.59‐, 1.27‐, 1.54‐, and 1.72‐fold of that in peptide group, respectively. Similar trends could be observed on protein level (Figure 3C,D]). With AGEs incubation, VEGFA and MMP9 were attenuated to 0.84‐ and 0.45‐fold of control group. They were upregulated to 1.27‐ and 0.94‐fold by p@tFNA, corresponding to 1.00‐ and 0.63‐fold in the peptide group.

As to the underlining mechanism, studies have shown the role of ERK1/2 phosphorylation in the healing peptide functioning process. In our work, we measured the levels of ERK1/2 and p‐ERK1/2 in addition to VEGFA and MMP9. The phosphorylation level of ERK1/2 was elevated to 1.49‐fold after p@tFNA induction (Figure 3E,F). To further verify the role of ERK1/2 phosphorylation during the proangiogenic regulation of p@tFNA function, we inhibited the phosphorylation of ERK1/2 and detected the subsequent variation in VEGFA expression and tube formation. Exposure to the inhibitor of ERK1/2 (LY3214996, Selleck, Shanghai, China) attenuated p‐ERK1/2 and VEGFA expression to 0.69‐fold and 0.59‐fold (Figure 3G,H]) even after treatment with p@tFNA. Decreased density and quantity of vessel‐like structures were also observed in Figure 3I,J. The nodes, junctions and meshes of the newly formed vessel‐like structures in the inhibitor+p@tFNA group still decreased to 0.20‐fold, 0.19‐fold, and 0.13‐fold. Parameters including total mesh area, total length, branching length and branching intervals decreased to 0.02‐fold, 0.35‐fold, 0.25‐fold, and 0.49‐fold, respectively.

### 3.5 Antioxidant ability and intracellular ROS scavenging activity of p@tFNA

**FIGURE 4:**
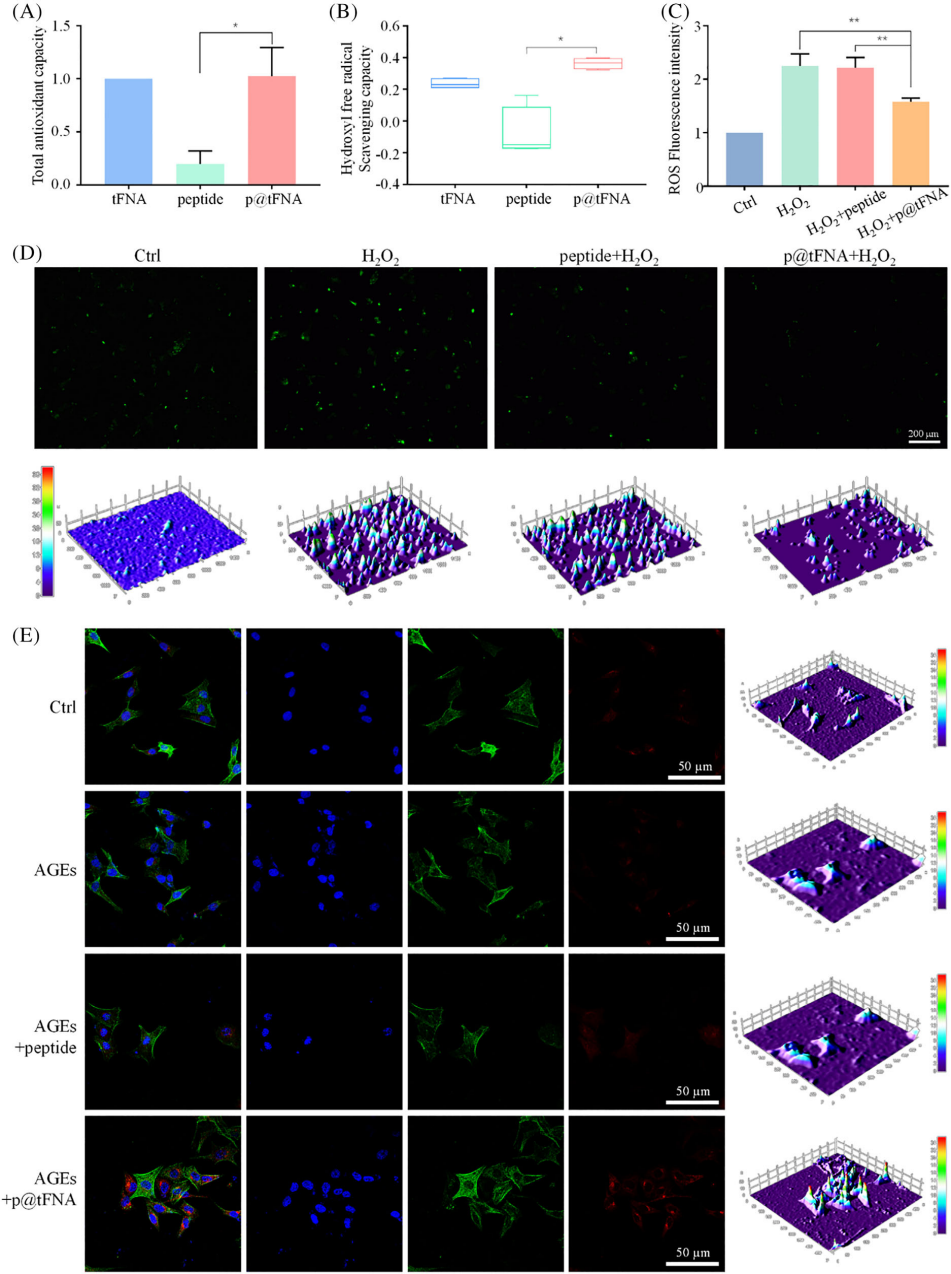
p@tFNA scavenged hydroxyl free radicals and intracellular ROS. (a) Total antioxidant capacity assay results of tFNA and p@tFNA. (b) Hydroxyl free radical scavenging capacity detection of tFNA and p@tFNA. (c) Fluorescence quantitative detection of ROS production with equalization by cell counting in control group, H_2_O_2_ group, H_2_O_2_ + peptide group and H_2_O_2_ + p@tFNA group. (e) Fluorescence microscope examinations and interactive 3D surface plot reconstruction of intracellular ROS levels of endothelial cells in the control group, H_2_O_2_ group, H_2_O_2_ + peptide group and H_2_O_2_ + p@tFNA group. (f) Immunofluorescence staining and interactive 3D surface plot reconstruction of HO‐1 expression in control group, AGEs group, AGEs+peptide group and AGEs+p@tFNA (600:1) group. Data are presented as mean ± SD (*n* = 4). Statistical analysis: **p < 0.01, *p < 0.05.

In our work, we examined the antioxidant ability of healing peptide, tFNA and p@tFNA to explore the influence of peptide on the inherent antioxidant ability of tFNA. The total antioxidant capacity (TAC) and hydroxyl free radical scavenging capacity assays revealed the ability to remove H_2_O_2_ and –OH, reflecting the antioxidant ability of the targeted reagent. The results in Figure 4A indicated that the moderate combination with peptide at 600:1 did not weaken the total antioxidant capacity of tFNA. The simple peptide exhibited little ability to eliminate O_2_‐ (0.20‐fold of that in simple tFNA group), whereas the elimination percentage in the p@tFNA group was up to 1.03‐fold of that in simple tFNA group. A similar trend could be observed in the hydroxyl free radical scavenging capacity assay (Figure 4B); compared with the 23.60% scavenging percentage in simple tFNA group, p@tFNA exhibited a slight advantage (up to 36.34%).

Reactive oxygen species assay kit was used to assess the in vitro cellular antioxidant application of p@tFNA. A labeled probe (DCFH‐DA) reacted with the active reactive oxygen species and the signal was quantified by fluorescence. We calculated the ratio of fluorescence values and cell number to obtain the value per cell and evaluated the intracellular ROS level. As shown in Figure 4C, intracellular ROS levels climbed sharply to 2.25‐fold of control group due to H_2_O_2_. In contrast, the application of p@tFNA attenuated ROS levels down to 1.58‐fold, more than simple peptide which had little influence on ROS (from 2.25‐fold to 2.22‐fold). Similar trends could be observed in the captures under a fluorescence microscope and corresponding interactive 3D surface plot analysis (Figure 4D). These ROS assays (Figure 4C,D) verified the in vitro antioxidant ability of p@tFNA. Furthermore, tFNA, as the frame core, retained integrality in the enzyme surroundings under protection of peptides, penetrated into the cell membrane, reached the cytosol, and exerted its antioxidant ability to cope with excessive ROS production.

Heme oxygenase‐1 (HO‐1), closely related to antioxidant activity, was stained and assessed in our work. As shown in Figure 4E, HO‐1 in the cytoplasm and nucleus was more obvious after exposure to p@tFNA than to simple peptide. 3D surface plot reconstruction and semi‐quantification showed that HO‐1 expression in p@tFNA group was 1.37‐fold of that in peptide group.

### 3.6 In vivo regenerative effect on skin defect

**FIGURE 5:**
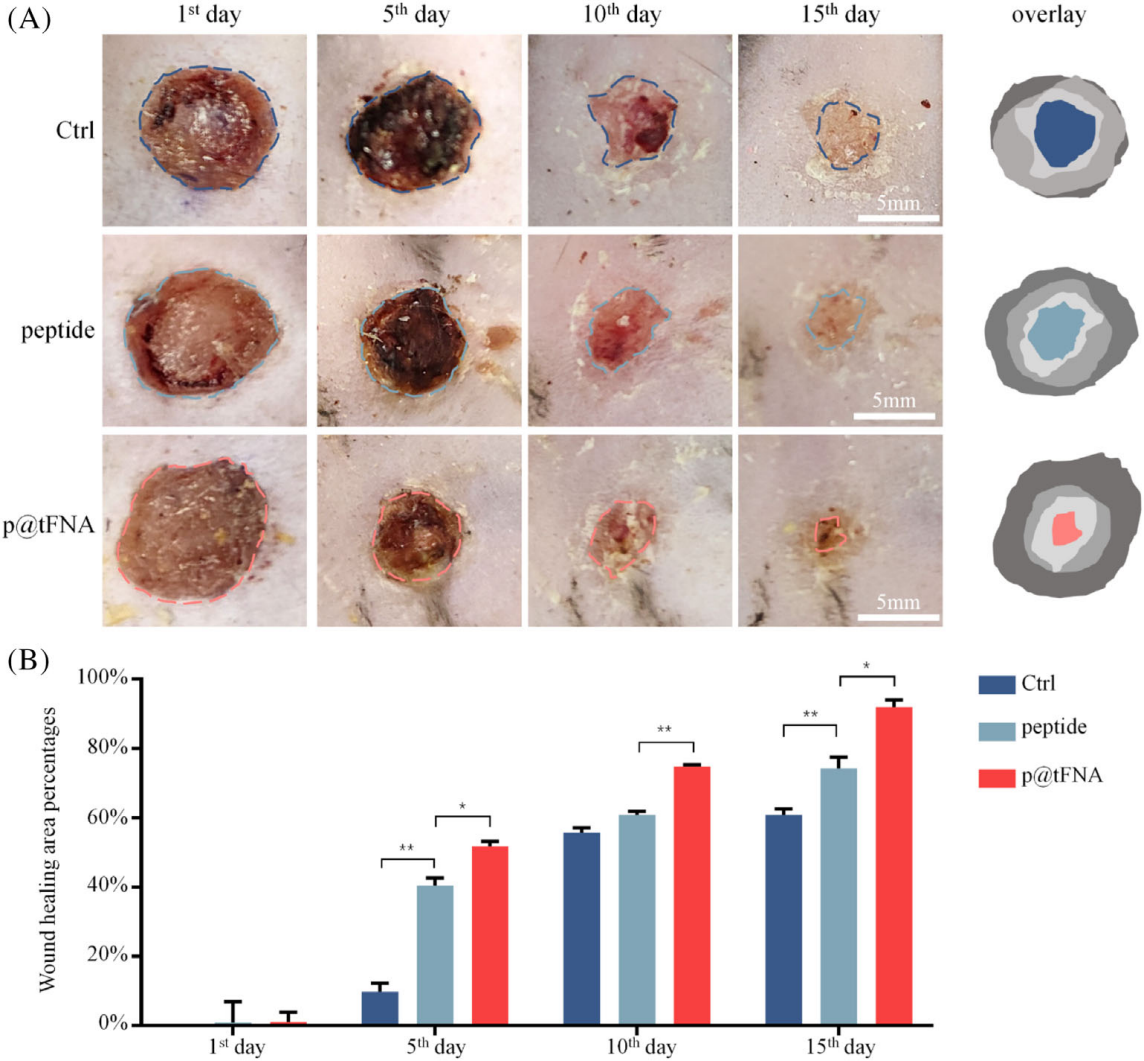
In vivo pro‐healing effect of p@tFNA on diabetic type II full‐thickness wounds. (a) Photographs of diabetic full‐thickness wounds (in db/db mouse) treated with PBS, peptide and p@tFNA at 1st, 5th, 10th, and 15th day from injection. (b) Measurement and analysis of the wound‐healing area percentages of each group. Data are presented as mean ± SD (*n* = 5). Statistical analysis: **p < 0.01, *p < 0.05.

Based on the better pro‐angiogenic performance of p@tFNA, we further evaluated whether p@tFNA could facilitate the full‐thickness wound closure in type II diabetes. Wounds were treated with (i) PBS, (ii) peptides, and (iii) p@tFNA. The healing process was tracked over 15 days. Wound closure was promoted in animals treated with p@tFNA during the first 5 days (Figure 5A,B). More specifically, the peptide group and p@tFNA group exhibited 40.49 ± 1.76% and 51.82 ± 1.11% wound closure on Day 5, respectively, which were obviously better than the PBS group (9.78 ± 2.02%). The p@tFNA group presented 5.27‐fold improvement in wound closure compared with the PBS group and 1.28‐fold improvement relative to the peptides. On Day 10 post‐wound, the wounds treated with p@tFNA improved to 74.79 ± 0.39% covering area while the peptide group reached 60.88 ± 0.78%. On Day 15, covering area of the p@tFNA group improved to 91.94 ± 1.71% and were nearly covered with newly formed tissue. In parallel, the peptide group exhibited a degree of healing activity to 74.20 ± 2.70% covering the area. The p@tFNA group exhibited 1.50‐fold wound closure efficiency of the PBS group and 1.23‐fold of the peptide group.

**FIGURE 6:**
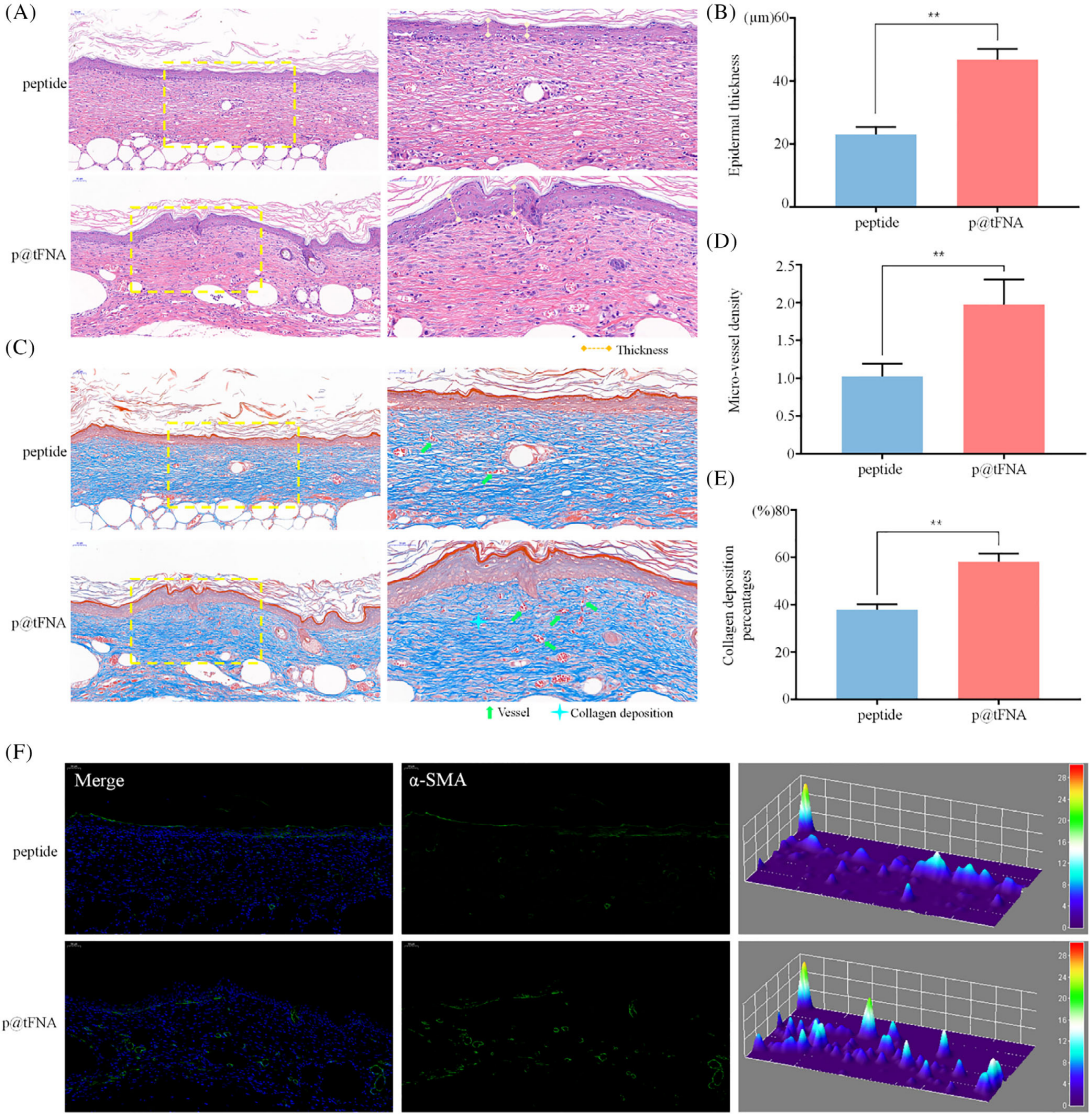
Histological evaluation of newly formed tissues during wound healing. (a) HE staining images of skin sections of the peptide group and p@tFNA group. (b) Statistical analysis of average epidermal thickness. (c) Masson's trichrome staining images of skin sections of the peptide group and p@tFNA group. (d) Relative ratios of micro‐vessel numbers between the peptide group and p@tFNA group. (e) Statistical analysis of collagen deposition percentages. (f) Immunofluorescence staining and interactive 3D surface plot reconstruction of α‐SMA expression in the peptide group and p@tFNA group.

To further explore the wound healing ability at the tissue level, we adopted haematoxylin and eosin (H&E) and Masson staining at Day 15(Figure 6A,C). Indicators including epidermis thickness, vessel density and collagen deposition percentage were measured in Figure 6bD,E. In the p@tFNA group, as the restoration process progresses, it formed a fully and connected thick (46.80 ± 3.08 μm) epithelial structure which is similar to the healthy epidermis of normal skin. In comparison, the epidermis thickness in the peptide group was 23.08 ± 2.11 μm. Regenerated tissue in p@tFNA group also exhibited better vessel density and collagen deposition. The number of vessels observed in p@tFNA group was 1.97‐fold of those observed in peptide group. As shown in Masson's trichrome staining images (Figure 6C), with thicker granulation tissue, the wounds treated with p@tFNA deposited more collagen fibres (58.07 ± 3.13%) during newly formed tissue remodelling. In parallel, the peptide group exhibited a degree of 37.88 ± 2.02% collagen deposition. As one of the classic indicators of vessel structures, α‐SMA were also investigated in our work. Immunofluorescence staining and interactive 3D surface plot reconstruction of α‐SMA expression in Figure 6F further verified the better vessel density in p@tFNA group.

## 4 DISCUSSION

The intracellular application of healing peptides brings facilitation to better wound healing. However, enzymatic surroundings largely limit the stability, affect the bioavailability, and shorten the half‐life of peptides.^17^, ^19^ A facile and robust solution to penetrate cell membrane and protect cytosolic peptides from complicated surrounding interference remained in great demand. In consideration of its intrinsic programmability, stability, and biocompatibility, functional DNA‐based nanostructures acting as frameworks for drug incubation or functional group decoration are defined as framework nucleic acids (FNAs).^30^, ^31^, ^32^, ^33^ As a simple and classic architecture in the DNA nanostructure category, tetrahedral framework nucleic acids (tFNAs) are characterized by concise fabrication, high yield, better cellular uptake, size‐endowed tissue penetration, and low toxicity. Previous studies also indicated that tFNA helps scavenge reactive oxygen species, enhancing anti‐inflammatory effects and facilitating cell‐material interactions.^34^ Combined with the aforementioned intrinsic properties, these features define tFNA as a potent candidate for complex drug delivery systems. In this work, we developed a tFNA‐based peptide delivery system that enhances the transport efficiency of peptides and the stability of both tFNA and peptides.

Results in Figure 1 verified satisfactory synthesis of tFNA and smooth construction of p@tFNA. The electrostatic adsorption of healing peptide with positive charge slightly magnified the size of tFNA framework and partially neutralize its potential without influencing the inherent molecular weight of tFNA. In the composite structure of p@tFNA, the mutual protection against enzyme environment was distinctly recognizable. Combined with the facilitated cellular uptake, the tFNA acted as a framework carrier to support, protect and deliver healing peptide to cytosol. In turn, peptide encapsulation covered the surface of tFNA and helped resisting to digestion.

A crucial aspect of non‐healing nature of diabetic foot ulcers is the reduced angiogenesis through the whole phases.^35^ The excessive AGEs accumulation leads to disordered endothelial metabolism, extracellular matrix alteration and cytokine expression deregulation. Combined with oxidative stress evoked by AGEs, the EC proliferation and newly formed vessels were limited.^36^ Treatments should be designed and evaluated in the diabetic environments closer to compromised human wound healing rather than just normal condition.^35^ In our present work, p@tFNA was proved to enhance critical processes during vessel growth including migration, proliferation and tube information of ECs in AGEs inducing diabetic environment. The results mean that efficient delivery of healing peptide by tFNA helps overcome the angiogenic obstacles induced by AGEs and exhibits application prospect in wound healing. Better angiogenesis under diabetic paves the way for immune response, nutrition delivery and matrix remolding during the whole healing phases to achieve satisfactory regeneration.

Expression variations of VEGF, FGF, TGF‐β and PDGF also verified the positive role of p@tFNA on angiogenic processes. As a key angiogenic factor, VEGF tips scales at proangiogenic signaling transduction to angiogenesis processes.^37^ Apart from activating cell proliferation of ECs, the VEGF pathway loosens cell–cell contacts and degrades the ECM to promote endothelial cell migration and vascularization.^38^, ^39^ During the consecutive steps of angiogenesis, FGFs take effect by paracrine and autocrine mechanisms from endothelial and stromal cells.^38^ They bind to receptors and induce the activation of angiogenic downstream signalling cascades involved in proliferation, survival, migration, proteinase production, and endothelial cell barrier integrity.^40^ During later vascular remodelling, TGF‐β and PDGF expression help stabilize the mature vascular network through producting ECM and attracting pericytes and SMCs to form surroundings. During the process of vessel formation, MMP‐2 and MMP‐9 involves in remoulding ECM, liberating ECs and pericytes to shape a pro‐angiogenic environment.^41^

As to the underlying molecular mechanism during the function of p@tFNA, inhibition of ERK1/2 phosphorylation (Figure 3e–j) attenuated the angiogenic function of p@tFNA. Results of VEGFA expression and tube formation revealed the key role of ERK1/2 phosphorylation in p@tFNA enhancing angiogenesis. The ERK1/2 phosphorylation has been indicated by studies to promote cellular responses, including proliferation, migration and proangiogenic behaviors.^42^ Regarding proliferation, ERK signaling stimulates transcriptional induction of CCND1 and thus accelerates the G1/S transition.^42^ Furthermore, the activation of RAS–ERK is involved in motility program initiation induced by external signals and subsequent coordinated movement. The increased activation of ERK signalling also upregulated the production of VEGF and the response to VEGF.^43^, ^44^ The intracellular delivery of healing peptide with tFNA enhanced the ERK1/2 phosphorylation and subsequently facilitated the proliferation, migration and tube formation during angiogenesis.

Apart from angiogenic ability, anti‐oxidation exhibited in p@tFNA also pave the way for diabetic wound healing. Studies have illustrated that chronic diabetic wounds with hyperglycemia exhibit oxidizing microenvironment infiltration, inhibited antioxidant enzyme activity, and refractory wound closure.^45^, ^46^ With AGEs binding, excessive production of ROS oxidizes and modifies biological macromolecules, including DNA, lipids, and proteins.^4^, ^7^ In terms of DNA bases or double helix breaks induced by DNA oxidation, hydroxyl radical stress directly breaks pyrimidine, purine bases and strand excision of the DNA sugar‐phosphate backbone.^47^, ^48^ Hydroxyl radicals cause hydrogen abstraction and subsequently form ribose fragments. Mutagenic or clastogenic DNA fragmentation disturbs the compaction and coiling of DNA within chromatin and ultimately leads to genome dysfunction.^8^, ^49^

In view of above‐mentioned micro‐environment, delivery platforms with intrinsic anti‐inflammatory and antioxidant properties are deemed promising candidates for reinforced effects to address diabetic wound.^50^, ^51^ Studies have indicated the antioxidant ability of DNA due to its deoxyribonucleotide nature.^52^, ^53^ As a classic three‐dimensional structure of DNA, tFNA exhibited better cellular uptake capacity to deliver antioxidants (including itself) into target cells and organs.^54^ tFNA may act as a substitute substrate of DNA or RNA to react with excessive intracellular ROS, achieve intracellular ROS balance, and subsequently prevent the corresponding DNA damage response. The three‐dimensional structure of tFNA offers a larger contact zone for the neutralization reaction of free radicals. The moderate combination with healing peptides further protected the stability of tFNA for better cellular antioxidant application.

In the in vivo diabetic model, the faster wound healing rate could be observed after p@tFNA treatment. H&E, Masson and α‐SMA staining indicated vessel formation, collagen deposition and newly formed granulation tissue. During the proliferation phase, newly formed granulation tissue comprises well‐organized connective tissues and tiny blood vessels. It serves as a scaffold for wound healing process.^35^, ^55^ The length of epithelium, collagen deposition percentage and vessel density are crucial elements paving the way for wound closure. Combined with analysis in Figures 5 and Figure 6, the p@tFNA facilitated epidermis covering, vessel formation and collagen deposition and subsequently formed the foundation for better regenerative effect on skin defect.

## 5 CONCLUSION

In this study, we developed a tFNA‐based peptide delivery system that enhances the transport efficiency of peptides and the stability of both tFNA and peptides. First, more healing peptides reached the cytosol due to the excellent cell‐entrance ability of tFNA. Second, the peptides were protected on the basis of frame core. In turn, tFNA, as the core coated by peptides, exhibited better enzyme resistance ability. Third, the dimensional structure of DNA and abundant reaction sites for free radicals formed the foundation of antioxidant ability. Finally, better transportation of peptides triggered more ERK1/2 phosphorylation and subsequent downstream reactions involved in growth factor expression, proliferation, migration, and tube formation. This peptide delivery system can exert its effect on tissue regeneration, antibacterial therapy, and inflammatory diseases depending on the concrete peptide.

## AUTHOR CONTRIBUTIONS

The authors confirm contribution to the paper as follows: study conception and design: Shiyu Lin, Xiaoxiao Cai, Huiming Wang; Experiment and data collection: Shiyu Lin, Qi Zhang, Songhang Li; Analysis and interpretation of results: Shiyu Lin, Qi Zhang, Xin Qin; Draft manuscript preparation: Shiyu Lin, Qi Zhang, Songhang Li. All authors reviewed the results and approved the final version of the manuscript.

## CONFLICT OF INTEREST

The authors declare no conflict of interest.

## Data Availability

The data that support the findings of this study are available from the corresponding author upon reasonable request.
